# The associations between metabolic profiles and sexual and physical abuse in depressed adolescent psychiatric outpatients: an exploratory pilot study

**DOI:** 10.1080/20008066.2023.2191396

**Published:** 2023-03-29

**Authors:** Karoliina Kurkinen, Olli Kärkkäinen, Soili M. Lehto, Ilona Luoma, Siiri-Liisi Kraav, Petri Kivimäki, Anni I. Nieminen, Katriina Sarnola, Sebastian Therman, Tommi Tolmunen

**Affiliations:** a Institute of Clinical Medicine, University of Eastern Finland, Kuopio, Finland; b School of Pharmacy, University of Eastern Finland, Kuopio, Finland; c Institute of Clinical Medicine, University of Oslo, Oslo, Norway; d R&D Department, Division of Mental Health Services, Akerhus University Hospital, Lørenskog, Norway; e Department of Psychiatry, Faculty of Medicine, University of Helsinki, Helsinki, Finland; f Department of Child Psychiatry, Kuopio University Hospital, Kuopio, Finland; g Department of Social Sciences, University of Eastern Finland, Kuopio, Finland; h Metabolomics Unit, Institute for Molecular Medicine Finland (FIMM), University of Helsinki, Helsinki, Finland; i Department of Adolescent Psychiatry, Kuopio University Hospital, Kuopio, Finland; j Mental Health Team, Finnish Institute for Health and Welfare, Helsinki, Finland

**Keywords:** Abuse, depression, metabolomics, psychiatry, trauma, Abuso, depresión, metabolómica, psiquiatría, trauma, 滥用, 抑郁, 代谢组学, 精神病, 创伤

## Abstract

**Background**: Sexual and physical abuse have been associated with long-term systemic alterations such as low-grade inflammation and changes in brain morphology that may be reflected in the metabolome. However, data on the metabolic consequences of sexual and physical abuse remain scarce.

**Objective**: This pilot study sought to investigate changes in the metabolite profile related to sexual and physical abuse in depressed adolescent psychiatric outpatients.

**Method**: The study included 76 patients aged 14–18 years, whose serum samples were analysed with a targeted metabolite profiling methodology. We estimated the associations between metabolite concentrations and the Trauma and Distress Scale (TADS) Sexual and Physical Abuse factor scores using three linear regression models (one unadjusted and two adjusted) per metabolite and trauma type pair. Additional variables in the two adjusted models were 1) the lifestyle indicators body mass index, tobacco use, and alcohol use, and 2) depression scores and the chronicity of depression.

**Results**: TADS Sexual Abuse scores associated positively with homogentisic acid, as well as cystathionine, and negatively with choline in linear regression analysis, whereas TADS Physical Abuse scores associated negatively with AMP, choline, γ-glutamyl cysteine and succinate, and positively with D-glucuronic acid.

**Conclusions**: This pilot study did not include a healthy control group for comparison and the cohort was relatively small. Nevertheless, we observed alterations in metabolites related to one-carbon metabolism, mitochondrial dysfunction, oxidative stress, and inflammation in depressed patients with a history of sexual or physical abuse.

## HIGHLIGHTS

- Metabolomic profiles associate with sexual or physical abuse.
- Metabolites relate to mitochondria, one-carbon, oxidative stress, and inflammation.
- Metabolomics a possible tool for precision psychiatry in the future.

## Abstract (es)

**Antecedentes:** El abuso sexual y físico se ha asociado con alteraciones sistémicas a largo plazo, tales como inflamación de bajo grado y cambios en la morfología cerebral que pueden reflejarse en el metaboloma. Sin embargo, los datos sobre las consecuencias metabólicas del abuso sexual y físico siguen siendo escasos.

**Objetivo:** Este estudio piloto buscó investigar los cambios en el perfil de metabolitos relacionados con el abuso sexual y físico en pacientes ambulatorios psiquiátricos adolescentes deprimidos.

**Método:** El estudio incluyó a 76 pacientes de 14 a 18 años, cuyas muestras de suero se analizaron con una metodología de perfil de metabolitos específicos. Estimamos las asociaciones entre las concentraciones de metabolitos y las puntuaciones de los factores de abuso sexual y físico de la Escala de Trauma y Angustia (TADS en sus siglas en inglés) utilizando tres modelos de regresión lineal (uno sin ajustar y dos ajustados) por par de metabolitos y tipos de trauma. Las variables adicionales en los dos modelos ajustados fueron 1) los indicadores de estilo de vida, el índice de masa corporal, el consumo de tabaco y el consumo de alcohol, y 2) las puntuaciones de depresión y la cronicidad de la depresión.

**Resultados:** Las puntuaciones de abuso sexual de la TADS se asociaron positivamente con el ácido homogentísico, así como con la cistationina, y negativamente con la colina en el análisis de regresión lineal, mientras que las puntuaciones de abuso físico de la TADS se asociaron negativamente con AMP, colina, g-glutamil cisteína y succinato, y positivamente con D- ácido glucurónico.

**Conclusiones:** Este estudio piloto no incluyó un grupo de control sano para la comparación y la cohorte fue relativamente pequeña. Sin embargo, observamos alteraciones en metabolitos relacionados con el metabolismo de un carbono, disfunción mitocondrial, estrés oxidativo e inflamación en pacientes deprimidos con antecedentes de abuso sexual o físico.

## Abstract (zh)

**背景：**性和身体虐待与长期的系统性改变有关，例如低度炎症以及可能反映在代谢组中的脑形态改变。 但是，关于性和身体虐待的代谢后果的数据仍然很少。

**目的：**本初步研究试图考查青少年精神病门诊病人中与性和身体虐待有关的代谢物概况改变。

**方法：**本研究纳入了76名14至18岁的患者，通过靶向的代谢物分析方法分析其血清样品。我们对于每种代谢物和创伤类型配对组使用三个线性回归模型（一个未调整和两个有调整的），估计了代谢物浓度与创伤和痛苦量表（TADS）性和身体虐待因子评分之间的关联。两个调整后的模型中的其他变量是1）生活方式指标，体重指数，烟草使用和酒精使用，以及2）抑郁评分和抑郁的长期性。

**结果：**TADS性虐待评分在线性回归分析中与尿黑酸以及胱硫醚正相关，与胆碱负相关，而TADS身体虐待评分与AMP、胆碱、G-谷氨酸半胱氨酸和氧化物和D-呈负相关，与D- 葡萄糖酸呈正相关。

**结论：**本初步研究未纳入健康对照组做对比，并且队列相对较小。 然而，我们观察到与具有性或身体虐待史的抑郁患者中与单碳代谢，线粒体功能障碍，氧化应激和炎症有关的代谢物改变。

## 1. Introduction

Sexual and physical abuse are considered traumatic life events that may lead or predispose to various psychiatric disorders, such as major depressive disorder (MDD), or post-traumatic stress disorder (PTSD; Adams et al., 2018). However, it is unclear how exposure to traumatic life events leads to the development of these disorders. The effects of trauma may be mediated or moderated by differences or changes in brain chemistry. For example, factors such as elevated low-grade inflammation (Michopoulos et al., 2017), or lowered levels of γ-aminobutyric acid (GABA) in plasma before trauma exposure have been suggested to increase the risk of or susceptibility to developing PTSD (Vaiva et al., 2006).

Childhood sexual and physical abuse have been associated with a higher prevalence of MDD (Levitan et al., 1998; Mandelli et al., 2015; Rohde et al., 2008). The lifelong consequences of childhood sexual and physical abuse are often considered severe (Adams et al., 2018; Guina et al., 2018), and they are intercorrelated in their severity, onset, and duration (Adams et al., 2018). A history of sexual abuse is often associated with an even longer duration of symptoms, such as avoidance and intrusive memories of trauma, than a history of physical abuse (Müller et al., 2018).

Beyond symptomatology, traumatic life events have been found to alter brain functions and metabolism (Ramage et al., 2016). For example, heightened responsivity of the amygdala and impaired functioning of the hippocampus have been observed in patients with PTSD (Shin et al., 2006). Even in clinically healthy subjects, experiences of childhood sexual abuse have been associated with neurocognitive abnormalities, such as poorer memory (Navalta et al., 2006). Furthermore, a large-scale cohort study utilising genomic and metabolomic data observed that the levels of citrate and glycoprotein acetyls affected the emotional and behavioural response to traumatic stress (Carvalho et al., 2020). A similar effect was observed for very-low-density lipoproteins (VLDL), both the level of large VLDL and total cholesterol in medium particles of VLDL (Carvalho et al., 2020). Indeed, PTSD has been suggested to associate with cardiometabolic dysfunction, changes in body mass index (BMI), and levels of creatinine, insulin, and glucose (Aliev et al., 2020).

Alterations in one-carbon metabolism have been suggested in psychological trauma (De Vries et al., 2015), and elevated levels of homocysteine have been recorded in PTSD patients when compared to healthy controls (Levine et al., 2008). In addition, mitochondrial dysfunction has been observed both after traumatic stress and in PTSD (Carvalho et al., 2020; Mellon et al., 2018). Sexual abuse early in life has been specifically associated with a decrease in the levels of antioxidants (Moraes et al., 2018), and increased oxidative stress has been associated with PTSD and physical neglect (Erjavec et al., 2018; Moraes et al., 2018). Lastly, disruptions in hypothalamic–pituitary–adrenal (HPA) axis activity or inflammatory cytokines have been found in PTSD (Kim et al., 2020; Michopoulos et al., 2017). Trauma, especially when experienced in childhood, is thus associated with an extensive variety of changes in metabolism and brain function.

Although sexual and physical abuse are often associated with psychiatric problems later in life, it is unclear to what degree there are observable biological changes due to these adverse experiences, or what metabolic alterations specific types of traumatic life events are associated with. However, some preliminary findings have been reported. Notably, disrupted stress regulation systems have been associated with childhood sexual abuse (Bellis et al., 2011). For example, a dysregulated HPA axis has been observed after both sexual and physical abuse (De Bellis & Zisk, 2014). To expand on these results, the present study investigated the associations between sexual and physical abuse traumatisation and a large variety of potential metabolic changes. In order to include a higher percentage of individuals with a history of trauma and thus increase the reliability of our analyses, we focused on a depressed population, in which the prevalence of traumatic events is enriched compared with the general population (Widom et al., 2007). Therefore, the aim of this pilot study was to investigate serum metabolite concentrations in depressed adolescents and young adults to improve our understanding of the associations between altered metabolic processes and sexual and physical abuse.

## 2. Methods

### 2.1. Study population

The current study formed part of the Systemic Metabolic Alterations Related to Different Psychiatric Disease Categories in Adolescent Outpatients (SMART) project, which recruited patients aged 14–20 years referred to the Adolescent Psychiatry Outpatient Clinic at Kuopio University Hospital (KUH) in the years 2017–2019. During this period, 445 were recruited and 192 of them gave blood samples. Of this subsample, 76 were diagnosed with MDD (*n* = 33; DSM-IV 296.20–296.36) or dysthymia (*n* = 12; DSM-IV 300.4), or both (*n* = 31), using the Structured Clinical Interview for DSM-IV (SCID; First et al., 2002), and were further dichotomised into MDD or chronic depression (dysthymia or double depression). All participants gave written informed consent and completed the research protocol. The SMART project complies with the Declaration of Helsinki (World Medical Association, 2013) and was approved by the Research Ethics Committee of the KUH in 2017.

### 2.2. Questionnaires and clinical assessments

Depressive symptoms were assessed with the Beck Depression Inventory (BDI; Beck et al., 1979). The BDI measures physical symptoms, behaviour, cognition, and feelings with 21 items scored 0–3 for a sum score range from 0 to 63. The first three questions of the Alcohol Use Disorders Identification Test (AUDIT; Saunders et al., 1993), focusing on consumption, were used to estimate the patients’ drinking habits on a scale from 0 to 12. The Alcohol, Smoking and Substance Involvement Screening Test (ASSIST 3.1; Humeniuk et al., 2010) was used to evaluate the patients’ smoking habits on a scale ranging from 0 to 31. A modified set of 16 questions from the Index of Diet Quality (IDQ; Leppälä et al., 2010) was used to estimate the health-promoting features and quality of the patients’ diet. In the short version of the IDQ, the response options were the dichotomous ‘never or nearly never’ or ‘yes, always or nearly always’ instead of a rating scale. The current medications of the patients were also inquired about and placed into the following six categories: 5. Antipsychotic medication (*n* = 13), 4. Selective serotonin reuptake inhibitors (SSRI; *n* = 15), 3. Mirtazapine (*n* = 3), 2. Agomelatine, tricyclic antidepressants, or vortioxetine (*n* = 4), 1. Other medications (melatonin, mini-pill, or oxazepam; *n* = 10), or 0. No medication (*n* = 31). The patients were grouped based on their medication to exclude bias caused by the use of different drug classes. The use of SSRI medication was evaluated also separately: 25 patients out of 76 were taking SSRI medication. The Sexual Abuse and Physical Abuse subscales of the Trauma and Distress Scale (TADS; Patterson et al., 2002; Salokangas et al., 2016) were used to assess lifetime sexual and physical abuse-related trauma, comprising recurring and nonrecurring traumatic life events as continuous scales. The effects of chronic depression and MDD were assessed with a dichotomous variable, as the patients were suffering from either episodic MDD or chronic depression, with the most recent SCID diagnosis extracted from the medical records.

### 2.3. Blood sampling

Blood was sampled in the morning after 12 h of fasting. Samples rested for 30 min and were then centrifuged at 2500 × *g* for 10 min. Centrifuged samples were prepared, and the serum was stored at −70 °C. After collecting all the samples, analyses were performed in one batch. Sample collection and storage was conducted by the laboratory unit ISLAB at KUH.

### 2.4. Targeted metabolomics analysis

Metabolomics analyses were conducted at the Institute for Molecular Medicine Finland. High-performance liquid chromatography coupled to mass spectrometry (HPLC-MS) was implemented for targeted metabolomics analysis. Altogether, 100 µL of serum was mixed with 10 µL of isotopically labelled internal standard, and the resulting mixtures were allowed to equilibrate. Supernatant was formed by adding 400 µL of extraction solvent (acetonitrile, 1% formic acid) and collected. The supernatant of the samples was transferred to a 96-well microplate and filtered on a Hamilton On-Deck Vacuum Station (300–400 mbar, 2.5 min). Five µL of each sample was then injected into a HPLC-MS system (Xevo® TQ-S triple quadrupole mass spectrometer, Waters Corporation, Milford, MA, USA). HPLC-MS was operated with positive and negative polarisation switching every 20 milliseconds for isolation and measurement of the metabolites. The measurement was conducted in Multiple Reaction Monitoring (MRM) accession mode. MassLynx 4.1 software was employed for data collection and for handling and management of the instrument, and TargetLynx 4.1 for data processing. Aspartate, cGMP, folic acid, homoserine, 5-hydroxyindole-3-actic acid, 3-OH-DL-kynurenine, orotic acid, pyridoxine, and sorbitol were not used in the final statistical analyses due to failed linearity or poor quality of the chromatograph, and UDP-glucose was not included due to missing 99% of values, resulting in 92 metabolite concentrations in total.

### 2.5. Statistical analysis

The SMART data set (*n* = 445) was used to estimate a confirmatory item factor model of the five *a priori* TADS factors with Mplus 8.3 software (Muthén & Muthén, 2017) using the WLSMV estimator, theta parameterisation, and default settings. The pairwise coverage between the 25 items was 97.7% at its lowest and 98.6% on average, making the impact of missingness minimal under the missing at random assumption. The model fit was acceptable: CFI .974, RMSEA .052, and SRMR .057. Standardised factor loadings and response thresholds are presented in Supplementary Table 3. Factor scores for the Physical Abuse and Sexual Abuse scales were calculated with the maximum *a posteriori* method for use in further analyses.

Linear regression analyses were run to analyse the association between TADS Sexual/Physical Abuse scores (*n* = 76) and the individual measured metabolites, separately for each trauma type and metabolite pair. In addition to these unadjusted models, the following background variables with possible effects on metabolism were used as covariates: gender, age, BMI, eating habits (IDQ), ongoing medications, depression symptom levels (BDI), depression chronicity, smoking (ASSIST tobacco), and alcohol drinking habits (AUDIT-C). Covariates for the analyses were chosen due to being associated with Sexual or Physical Abuse factor scores, respectively, in linear regressions at the .01 significance level, and to avoid overfitting, these covariates were divided into two separate models for each metabolite. Model 1 was adjusted for the effect of the participants’ lifestyle on the metabolome, with BMI, ASSIST Tobacco, and AUDIT-C scores being taken into consideration. Model 2 was adjusted for depressive symptomology, considering BDI scores and the chronicity of depression.

In addition to predicting trauma levels separately for each metabolite concentration, two multivariate regression analyses were conducted, one for each trauma type. For these multivariate analyses, metabolite concentration data were consolidated with principal component analysis (PCA) using SIMCA (version 17; Sartorius Stedim Data Analytics AB). To take multiple testing into consideration, we adjusted the level of *α* by dividing it with the principal component count explaining 95% of the variation in the metabolomics data in the PCA. This method was used due to the correlative nature of metabolites in the targeted metabolite profiling analyses (Würtz et al., 2016). The observed differences in *p*-values between .05 and the adjusted *α* were regarded as trends.

## 3. Results

### 3.1. Associations between trauma variables and demographic and other clinical variables

The demographic and clinical characteristics of the study cohort, such as age, gender, and symptom levels, are presented in Table 1, along with their unstandardised coefficients in the unadjusted regression model in which they were included. TADS Sexual Abuse factor scores were positively associated with depression levels and AUDIT-C scores, whereas Physical Abuse factor scores were positively associated with ASSIST Tobacco scores (Table 1). Both sexual and physical abuse were negatively correlated with the chronicity of depression, indicating a higher incidence of episodic depression in traumatised patients.

**Table 1.** Characteristics of the participants with covariates in the multivariate models, and linear regression coefficients in models predicting Trauma and Distress Scale (TADS) Sexual Abuse and Physical Abuse factor scores.

Demographic or clinical factor		TADS Sexual Abuse		TADS Physical Abuse	
		*B*	*p*	*B*	*p*
Male *n* (%)	12 (16)	.017	.887	-.155	.182
Age, mean (SD)	16.43 (1.57)	.194	.092	.071	.543
BMI, mean (SD)	23.33 (5.67)	.135	.245	.195	.091
IDQ scores, mean (SD)	25.61 (5.69)	-.199	.084	-.204	.077
Smoking, mean (SD)	6.49 (8.99)	.182	.116	.**258**	.**024**
Alcohol consumption, mean (SD)	2.70 (2.94)	.**24**	.**037**	.098	.398
BDI scores, mean (SD)	30.18 (7.57)	.**29**	.**011**	.129	.267
Chronic depression *n* (%)	43 (57)	**-**.**232**	.**044**	**-**.**329**	.**004**
Medication *n* (%)	45 (59)	-.048	.231	-.036	.346
SSRI *n* (%)	25 (33)	.034	.845	-.034	.838

Legend: Smoking (ASSIST Tobacco scale, Alcohol, Smoking and Substance Involvement Screening Test); Alcohol consumption (AUDIT-C, Alcohol Use Disorder Identification Test); BDI, Beck Depression Inventory; BMI, body mass index; IDQ, Index of Diet Quality subset; medication, including agomelatine, mirtazapine, SSRI, antipsychotic medication, or other medications; *B*, standardised regression coefficient; *p*, *p-*value from linear regression (significance); SD, standard deviation; SSRI, medication with only selective serotonin reuptake inhibitors.

### 3.2. Linear regressions predicting TADS factors with metabolite concentrations

Regression analyses demonstrated that some metabolites were associated with TADS Sexual and Physical Abuse. The linear regression between Sexual Abuse scores and metabolite concentrations showed a negative trend for choline (*p = *.004), and positive trends for cystathionine (*p = *.008) and homogentisic acid (*p = *.022). After implementing Model 1, adjusted for the patient’s lifestyle, choline, cystathionine, and homogentisic acid had *p-*values of .032, .018, and .04, respectively. After implementing Model 2, with adjustments for BDI scores and chronicity, cystathionine (*p = *.012) and homogentisic acid (*p = *.050) displayed a trend towards statistical significance, whereas choline (*p = *.268) had a stronger association with depressive symptoms and did not remain noteworthy (Supplementary Table 1).

The unadjusted linear regression between TADS Physical Abuse scores and metabolites revealed negative trends for choline (*p = *.016), AMP (*p = *.031), and succinate (*p = *.042), and a positive trend for D-glucuronic acid (*p = *.043). Out of these, succinate and D-glucuronic acid did not remain significant in Models 1 and 2. Choline (*p = *.009) remained significant in Model 1, adjusted for lifestyle factors, but not in linear regression Model 2, adjusted for BDI and depression chronicity. However, AMP remained significant in Model 2 (*p = *.009) but not in Model 1. Gamma glutamyl cysteine did not show an association in the linear regression alone, but after implementing Models 1 (*p = *.036) and 2 (*p = *.035), negative trends were seen between TADS Physical Abuse scores and γ-glutamyl cysteine (Supplementary Table 2).

### 3.3. Multiple testing with PCA

The results from PCA demonstrated that some metabolites were associated with TADS scores for sexual abuse and physical abuse. However, it should be noted that the principal components having the strongest association with abuse history (components 3 and 5) only explained 6% and 5% of the variance in the data, respectively. Furthermore, 42 components were required to describe 95% of the variation in the metabolomics data, so the *α*-level adjusted for multiple testing was set to .0012. No associations of metabolite concentrations and the abuse indicators were below this level.

## 4. Discussion

### 4.1. Main findings

**Figure 1.**
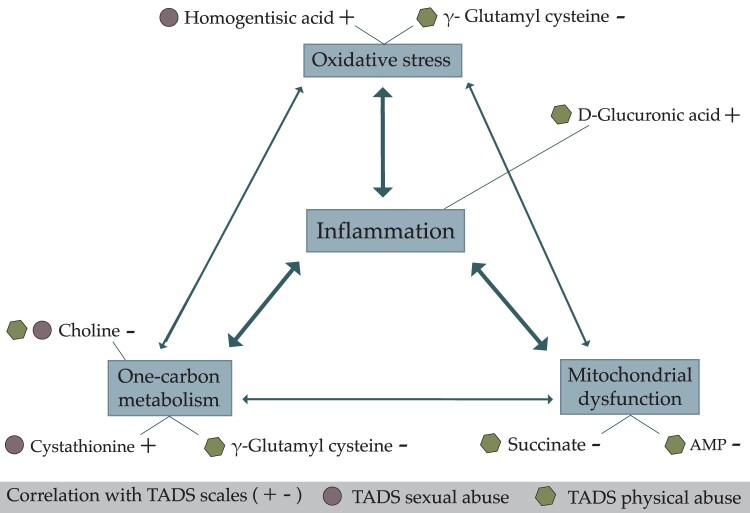
Schematic illustration of metabolites correlating positively (+) or negatively (-) with the Trauma and Distress Scale (TADS) Sexual and Physical Abuse scores, and the related systems or mechanisms in which these metabolites are involved.

This pilot study aimed to discover metabolic alterations in sexually or physically abused depressed adolescent psychiatric outpatients. The results of the present analysis point to altered processes in one-carbon metabolism, mitochondrial function, oxidative stress, and inflammation. These processes are also interconnected (Figure 1). Both sexual and physical abuse had a negative correlation with chronicity of depression, suggesting that episodic MDD is more common in these abused patients than dysthymia or double depression is.

### 4.2. One-carbon metabolism

**Figure 2.**
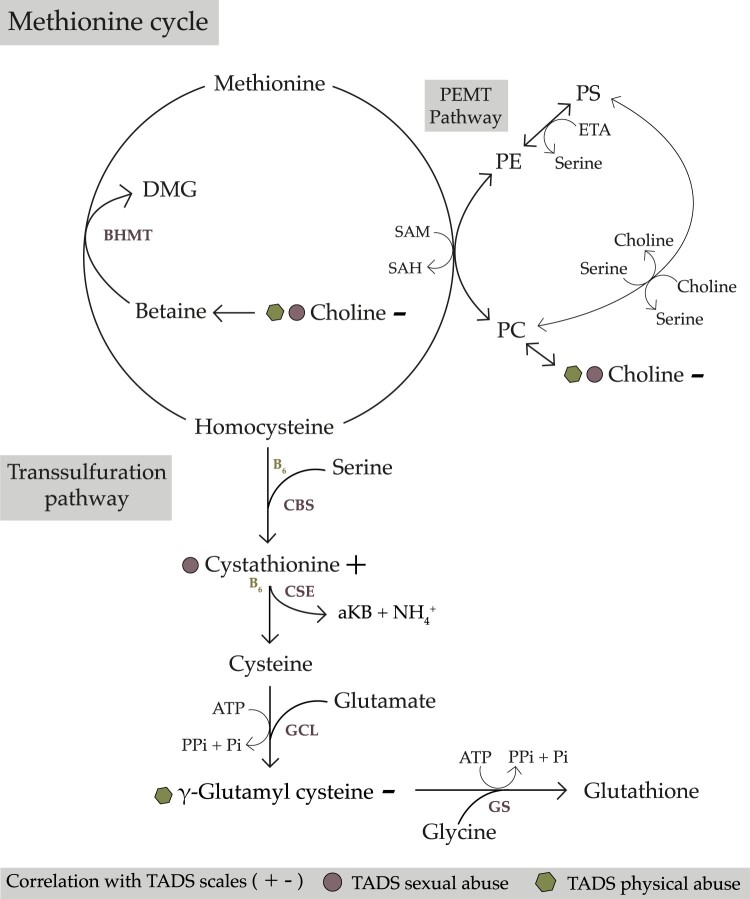
Illustration of the one-carbon metabolism methionine cycle, as well as the transsulfuration and phosphatidylethanolamine N-methyltransferase (PEMT) pathways. Round symbols represent the TADS Sexual Abuse scale, hexagons the TADS Physical Abuse scale, and + and – symbols represent the direction of correlation between the scale and the metabolite. aKB, α-ketobutyrate; ATP, adenosine triphosphate; BHMT, betaine-homocysteine S-methyltransferase; CBS, cystathionine β-synthase; CSE, cystathionine gamma-lyase; DMG, dimethylglycine; ETA, ethanolamine; GCL, glutamate-cysteine ligase; GS, glutathione synthetase; PC, phosphatidylcholine; PE, phosphatidylethanolamine; PEMT, phosphatidylethanolamine N-methyltransferase; Pi, phosphate; PPi, pyrophosphate; PS, phosphatidylserine; SAH, S-adenosylhomocysteine; SAM, S-adenosylmethionine.

Choline, γ-glutamyl cysteine and cystathionine take part in one-carbon metabolism, which has been found disrupted in PTSD (De Vries et al., 2015) and in other psychiatric disorders such as MDD (Kurkinen et al., 2021). One-carbon metabolism is an important regulatory factor in epigenetics, involved in the synthesis of phospholipids, delivering the functional properties of various proteins, and forming part of RNA metabolism (De Vries et al., 2015). Furthermore, neurotransmitters serotonin and noradrenaline require one-carbon metabolism in their synthesis (De Vries et al., 2015). However, choline did not remain significant when background factors related to depression type and intensity were controlled for. For this reason, the negative association of choline is possibly related to MDD in these patients (Table 1; Kurkinen et al., 2021). Furthermore, negative trend of γ-glutamyl cysteine and positive trend of cystathionine suggests that instead of the methionine cycle, the transsulfuration pathway of one-carbon metabolism is more relevant to the pathophysiology of trauma itself (Figure 2).

### 4.3. Mitochondrial dysfunction

Mitochondrial dysfunction could explain the negative correlation of AMP and succinate with TADS Physical Abuse scores in the present study. Animal studies have linked traumatic symptoms and intergenerational trauma to mitochondrial dysfunction (Alhassen et al., 2021; Preston et al., 2020). Furthermore, low succinate levels have been shown in MDD when compared to chronically depressed patients (Kurkinen et al., 2021), and mitochondrial dysfunction has been proposed as one possible pathological mechanism in PTSD (Bersani et al., 2020; Daniels et al., 2020). In the present study, succinate concentrations appeared to be related more to MDD than a history of physical abuse, since controlling for depression length and severity reduced the strength of the association (Supplementary Table 2). In addition, AMP lost its significance when lifestyle was considered.

### 4.4. Oxidative stress

Both homogentisic acid and γ-glutamyl cysteine, associating with physical and sexual abuse in this pilot study, have been suggested to have roles in oxidative stress (Ribas et al., 2014; M. L. Schiavone et al., 2020). Oxidative stress is elevated by chronic stress (Miller & Sadeh, 2014; Schiavone et al., 2013), and metabolomic and genetic studies have linked oxidative stress with PTSD (Alzoubi et al., 2019; Miller et al., 2018). It has been suggested that increased levels of γ-glutamyl cysteine might act as a compensatory mechanism against oxidative stress if the glutathione pathway is compromised (Ristoff et al., 2002). The formation of γ-glutamyl cysteine is the rate-limiting step in the glutathione pathway (Lu, 2013). γ-Glutamyl cysteine formation may have been disrupted, for instance, due to reduced glutamate cysteine ligase (GCL) enzymatic activity or accumulated glutamate interrupting cysteine influx or synthesis under oxidative stress (Zhu et al., 2022). Downregulation of the catalytic part of GCL has been associated with reduced glutathione in inflammation (Zhang et al., 2020). In this respect, the negative γ-glutamyl cysteine trend with physical abuse might indicate disruptions in the pathways against cellular oxidative stress. Homogentisic acid may act as an antioxidant or a pro-oxidant, depending on its cellular concentration and the cell type (Jurič et al., 2021; Kang et al., 2005; Rosa et al., 2011).

### 4.5. Inflammation

The trend towards elevated D-glucuronic acid levels in physically abused patients could reflect an increased level of inflammation, since inflammation appears to increase its circulating levels (Ho et al., 2019). D-glucuronic acid has been found to increase the activity of toll-like receptor 4 (Lewis et al., 2013), which is able to initiate an inflammatory cascade and induce systemic inflammation (Buchanan et al., 2010). Increased levels of D-glucuronic acid have been observed in severely physically traumatised patients, and the levels increased over time in patients who developed chronic critical illness (Horn et al., 2021). Changes in D-glucuronic acid might be more strongly associated with lifestyle and depression than trauma per se. However, glutathione produced in transsulfuration is not only part of one-carbon metabolism but is also connected to mitochondrial functions via oxidative stress, as well as inflammation. For example, glutathione has been suggested to modify the metabolic state of inflammatory T-cells (Mak et al., 2017), and similar T-cell redox alterations have been observed in animal studies modelling psychological trauma with Social Defeat Stress (Moshfegh et al., 2019).

### 4.6. Strengths and limitations

Our study provides new insights regarding metabolic events associated with trauma. The participants were on average 16 years old and therefore our findings represent traumatisation at a relatively early age. In turn, the cohort was quite small, reducing statistical power to detect differences in metabolite levels. Some of the identified alterations did not remain statistically significant after adjustment for background factors, and none of the changes remained statistically significant after correction for multiple testing. Differences in the metabolome related to chronic and episodic depression have been recognised in the previous literature, and some of the metabolic alterations observed in this study might therefore also be explained by MDD (Kurkinen et al., 2021). The study also lacked a healthy control group. Nevertheless, these alterations fit well in the framework that previous research has built for trauma and should be investigated further in larger cohorts. Furthermore, it was not considered that one individual might have a history of both sexual and physical abuse. We limited our study to sexual and physical abuse to reduce the number of analyses in this pilot study. Another limitation was the lack of information regarding when these abusive events occurred and their duration, although it is often the case that traumatic life events extend over a longer time period. However, TADS has been suggested to be a reliable tool to measure the level and type of childhood traumatisation, regardless of the limited knowledge of each particular traumatic event (Salokangas et al., 2016). Furthermore, genders were unevenly represented, as only one participant in six was male, which reflects depression, treatment-seeking, and study participation being more common in females. Limited statistical power did not allow us to perform subgroup analysis by gender. However, we selected all the participants with depressive disorder in the order of appearance, and there was therefore no systematic bias in gender distribution in our sample. Rather, the difference reflects the prevalence rates of depressive disorder in different genders. There are also hormonal differences between the genders. In the future, the effect of hormones on the metabolome could be controlled for with the steroidal hormone levels, especially in youths undergoing puberty. The use of peripheral blood samples is limited from the central nervous system point of view, although the events in the CNS are suggested to reflect in the periphery to some extent (Tylee et al., 2013), especially in the case of inflammation (Cervellati et al., 2020).

## 5. Conclusions

Our pilot study suggests that metabolites related to one-carbon metabolism, mitochondrial dysfunction, oxidative stress, and inflammation may be altered in sexually and physically abused depressed adolescent patients. When depression is considered, oxidative stress and the transsulfuration pathway of one-carbon metabolism are suggested as the most relevant mechanisms for trauma in this pilot study. Further research is needed to confirm these findings in larger and more diverse cohorts. In addition, follow-up samples and alternative omics techniques could be used to better understand the impacts of traumatic life events, for example, on gene expression as well as on the metabolome.

## Supplementary Material

Supplemental Material

Supplemental Material

Supplemental Material

Supplemental Material

## Data Availability

The data that support the findings of this study are available on request from the corresponding author, KK. The data are not publicly available due to their containing information that could compromise the privacy of research participants. The study plan approved by the ethical committee and the participant consent terms preclude public sharing of these sensitive data, even in anonymized form.
