# Supplementary material for: The associations between metabolic profiles and sexual and physical abuse in depressed adolescent psychiatric outpatients: an exploratory pilot study
Source: Eur J Psychotraumatol. 2023 Mar 29;14(1):2191396. doi: 10.1080/20008066.2023.2191396 (PMC10062226; doi:10.1080/20008066.2023.2191396)
Supplement: Supplemental Material [file ZEPT_A_2191396_SM0864.docx]

Supplementary Table 1, Linear regression coefficients with Trauma and Distress Scale (TADS) Sexual Abuse factor scores across metabolites, adjusting for selected covariates in Models 1 and 2.

|  |  | Unadjusted model | | | | |  | Adjusted model 1 | | | | | |  | Adjusted model 2 | | | | |
| --- | --- | --- | --- | --- | --- | --- | --- | --- | --- | --- | --- | --- | --- | --- | --- | --- | --- | --- | --- |
|  |  | *B* | *p* |  | 95% CI | |  | *B* | *p* |  | 95% CI | | |  | *B* | *p* |  | 95% CI | |
|  |  |  |  |  | Lower bound | Upper bound |  |  |  |  | Lower Bound | Upper bound | |  |  |  |  | Lower bound | Upper bound |
| **Amino acids and derivatives** | | | |  |  |  |  |  |  |  |  |  | |  |  |  |  |  |  |
|  | | | |  |  |  |  |  |  |  |  |  | |  |  |  |  |  |  |
| Aminoadipic acid | | -.163 | .159 |  | -.380 | .063 |  | -.212 | .066 |  | -.425 | .014 | |  | -.151 | .176 |  | -.359 | .067 |
| Aminoisobutyric acid | | -.065 | .574 |  | -3.758 | 2.099 |  | -.089 | .446 |  | -4.064 | 1.806 | |  | -.115 | .298 |  | -4.209 | 1.306 |
| Alanine |  | .017 | .883 |  | -.002 | .002 |  | 0 | .998 |  | -.002 | .002 | |  | .101 | .367 |  | -.001 | .003 |
| Arginine |  | -.033 | .775 |  | -.012 | .009 |  | -.095 | .416 |  | -.015 | .006 | |  | -.04 | .716 |  | -.012 | .008 |
| Asparagine |  | .034 | .769 |  | -.016 | .021 |  | .044 | .703 |  | -.015 | .022 | |  | .119 | .289 |  | -.008 | .027 |
| Asymmetric dimethylarginine | | -.107 | .357 |  | -.825 | .301 |  | -.103 | .376 |  | -.817 | .312 | |  | -.119 | .28 |  | -.824 | .242 |
| Citrulline |  | -.185 | .11 |  | -.045 | .005 |  | -.186 | .105 |  | -.045 | .004 | |  | -.232 | .036 |  | -.049 | -.002 |
| Creatine |  | .007 | .953 |  | -.007 | 0,007 |  | -.107 | .4 |  | -.01 | .004 | |  | -.028 | .8 |  | -.007 | .006 |
| Creatinine |  | -.056 | .631 |  | -.012 | .007 |  | -.075 | .52 |  | -.013 | .006 | |  | -.017 | .88 |  | -.01 | .008 |
| **Cystathionine** | | **.301** | **.008** |  | **4.102** | **26.686** |  | **.278** | **.018** |  | **2.49** | **25.888** | |  | **.271** | **.012** |  | **3.168** | **24.582** |
| Dimethylglycine | | -.071 | .542 |  | -.120 | .064 |  | -.092 | .424 |  | -.128 | .054 | |  | -.137 | .213 |  | -.141 | .032 |
| Glutamate | | -.038 | .744 |  | -.021 | .015 |  | -.138 | .258 |  | -.03 | .008 | |  | -.123 | .283 |  | -.028 | .008 |
| Glutamine |  | .096 | .407 |  | .000 | .001 |  | .074 | .534 |  | -.001 | .001 | |  | .157 | .158 |  | 0 | .001 |
| Glycine |  | .008 | .946 |  | -.001 | .001 |  | .02 | .867 |  | -.001 | .002 | |  | .039 | .726 |  | -.001 | .002 |
| Guanidinoacetic acid | | -.104 | .371 |  | -.352 | .133 |  | -.113 | .357 |  | -.374 | .137 | |  | -.02 | .856 |  | -.256 | .213 |
| Histidine |  | .042 | .716 |  | -.006 | .009 |  | .028 | .805 |  | -.006 | .008 | |  | .026 | .811 |  | -.006 | .008 |
| Homocysteine | | -.004 | .973 |  | -.864 | .835 |  | .037 | .75 |  | -.711 | .983 | |  | .008 | .943 |  | -.784 | .843 |
| **Homogentisic acid** | | **.262** | **.022** |  | **.352** | **4.480** |  | **.243** | **.04** |  | **.103** | **4.389** | |  | **.213** | **.05** |  | **-.003** | **3.941** |
| Hydroxyproline | | -.122 | .294 |  | -.038 | .012 |  | -.144 | .209 |  | -.04 | .009 | |  | -.099 | .406 |  | -.036 | .015 |
| Isoleucine |  | .143 | .219 |  | -.001 | .006 |  | .088 | .478 |  | -.003 | .005 | |  | .093 | .398 |  | -.002 | .005 |
| Kynurenic acid | | -.123 | .288 |  | -26.565 | 8.011 |  | -.138 | .25 |  | -28.176 | 7.441 | |  | -.089 | .426 |  | -23.23 | 9.917 |
| L-Kynurenine | | .036 | .756 |  | -.961 | 1.318 |  | .054 | .649 |  | -.894 | 1.426 | |  | .033 | .761 |  | -.905 | 1.233 |
| Leucine |  | .143 | .219 |  | -.002 | .010 |  | .11 | .372 |  | -.004 | .009 | |  | .085 | .444 |  | -.004 | .008 |
| Lysine |  | -.063 | .587 |  | -.006 | .004 |  | -.119 | .339 |  | -.008 | .003 | |  | -.048 | .664 |  | -.006 | .004 |
| L-Methionine | | .024 | .839 |  | -.020 | .024 |  | .023 | .84 |  | -.019 | .024 | |  | .055 | .615 |  | -.015 | .026 |
| Ornithine |  | .116 | .317 |  | -.006 | .018 |  | .05 | .673 |  | -.01 | .015 | |  | .081 | .461 |  | -.007 | .015 |
| Phenylalanine | | .146 | .209 |  | -.004 | .018 |  | .114 | .352 |  | -.006 | .017 | |  | .102 | .354 |  | -.005 | .015 |
| Proline |  | -.083 | .473 |  | -.004 | .002 |  | -.065 | .573 |  | -.004 | .002 | |  | -.079 | .475 |  | -.004 | .002 |
| Serine |  | -.011 | .923 |  | -.006 | .006 |  | .007 | .955 |  | -.006 | .006 | |  | -.004 | .969 |  | -.006 | .006 |
| Symmetric dimethylarginine | | -.012 | .92 |  | -.444 | .401 |  | 0 | .997 |  | -.417 | .419 | |  | -.052 | .634 |  | -.493 | .303 |
| Threonine |  | -.15 | .197 |  | -.006 | .001 |  | -.117 | .312 |  | -.006 | .002 | |  | -.096 | .386 |  | -.005 | .002 |
| Tryptophan |  | -.022 | .849 |  | -.051 | .042 |  | -.009 | .936 |  | -.048 | .044 | |  | .015 | .892 |  | -.041 | .047 |
| Tyrosine |  | .103 | .374 |  | -.009 | .023 |  | .078 | .503 |  | -.01 | .021 | |  | .107 | .328 |  | -.007 | .022 |
| Valine |  | .101 | .386 |  | -.001 | .002 |  | .068 | .578 |  | -.001 | .002 | |  | .074 | .498 |  | -.001 | .002 |
|  |  |  |  |  |  |  |  |  |  |  |  |  |  |  |  |  |  |  |  |
| **Choline and mitochondrial metabolites** | | | | | |  |  |  |  |  |  |  |  |  |  |  |  |  |  |
|  |  |  |  |  |  |  |  |  |  |  |  |  |  |  |  |  |  |  |  |
| Acetoacetic acid | | -.021 | .856 |  | -.001 | .001 |  | .038 | .746 |  | -.001 | .001 | |  | -.014 | .904 |  | -.001 | .001 |
| Allantoin |  | .076 | .516 |  | -.153 | .302 |  | .099 | .39 |  | -.127 | .322 | |  | .063 | .564 |  | -.152 | .276 |
| Glycine betaine | | -.051 | .659 |  | -.006 | .004 |  | -.044 | .7 |  | -.006 | .004 | |  | -.01 | .931 |  | -.005 | .005 |
| Carnitine |  | .05 | .667 |  | -.018 | .028 |  | .021 | .858 |  | -.021 | .025 | |  | .045 | .686 |  | -.017 | .026 |
| Carnosine |  | -.027 | .818 |  | -46.619 | 36.941 |  | -.038 | .742 |  | -47.995 | 34.334 | |  | -.061 | .581 |  | -50.511 | 28.536 |
| **Choline** |  | **-.236** | **.040** |  | **-.087** | **-.002** |  | **-.244** | **.032** |  | **-.088** | **-.004** | |  | -.128 | .268 |  | -.067 | .019 |
| Cotinine |  | .059 | .776 |  | -.533 | .706 |  | .237 | .262 |  | -.068 | .238 | |  | -.006 | .979 |  | -1.054 | 1.027 |
| Gamma-Glutamyl cysteine | | -.144 | .213 |  | -.295 | .067 |  | -.163 | .156 |  | -.308 | .05 | |  | -.153 | .161 |  | -.292 | .049 |
| Phosphoethanolamine | | -.114 | .326 |  | -.886 | .298 |  | -.102 | .374 |  | -.847 | .322 | |  | -.053 | .637 |  | -.703 | .433 |
| Spermidine |  | .04 | .732 |  | -3.282 | 4.649 |  | .025 | .835 |  | -3.59 | 4.431 | |  | .031 | .778 |  | -3.202 | 4.264 |
| Succinate |  | -.216 | .061 |  | -.092 | .002 |  | -.197 | .103 |  | -.09 | .008 | |  | -.175 | .124 |  | -.083 | .01 |
|  |  |  |  |  |  |  |  |  |  |  |  |  |  |  |  |  |  |  |  |
| **Acylcarnitines** | | | |  |  |  |  |  |  |  |  |  |  |  |  |  |  |  |  |
|  |  |  |  |  |  |  |  |  |  |  |  |  |  |  |  |  |  |  |  |
| Acetylcarnitine | | .075 | .52 |  | -.043 | .084 |  | .026 | .822 |  | -.056 | .07 | |  | -.001 | .993 |  | -.063 | .062 |
| Propionylcarnitine | | .109 | .35 |  | -12.053 | 33.610 |  | .043 | .728 |  | -20.054 | 28.552 | |  | .08 | .481 |  | -14.369 | 30.192 |
| Isobutyrylcarnitine | | -.007 | .954 |  | -17.733 | 16.729 |  | -.011 | .923 |  | -17.798 | 16.137 | |  | -.009 | .933 |  | -17.119 | 15.725 |
| Isovalerylcarnitine | | .18 | .12 |  | -2.509 | 21.394 |  | .136 | .263 |  | -5.45 | 19.681 | |  | .148 | .18 |  | -3.667 | 19.163 |
| Hexanoylcarnitine | | -.09 | .441 |  | -41.163 | 18.099 |  | -.082 | .491 |  | -41.056 | 19.903 | |  | -.085 | .453 |  | -39.549 | 17.835 |
| Octanoylcarnitine | | -.03 | .798 |  | -3.704 | 2.857 |  | .023 | .848 |  | -3.06 | 3.716 | |  | -.013 | .91 |  | -3.436 | 3.064 |
| Decanoylcarnitine | | -.006 | .958 |  | -6.034 | 5.723 |  | .054 | .65 |  | -4.605 | 7.331 | |  | -.018 | .877 |  | -6.24 | 5.34 |
|  |  |  |  |  |  |  |  |  |  |  |  |  |  |  |  |  |  |  |  |
| **Nucleotides and nucleosides** | | | |  |  |  |  |  |  |  |  |  |  |  |  |  |  |  |  |
|  |  |  |  |  |  |  |  |  |  |  |  |  |  |  |  |  |  |  |  |
| Deoxycytidine | | .109 | .35 |  | -.338 | .941 |  | .035 | .778 |  | -.583 | .776 | |  | .086 | .45 |  | -.387 | .863 |
| 2-Deoxyuridine | | -.046 | .693 |  | -11.562 | 7.721 |  | -.052 | .65 |  | -11.727 | 7.366 | |  | -.051 | .639 |  | -11.19 | 6.917 |
| Adenosine |  | -.034 | .768 |  | -.276 | .204 |  | -.04 | .728 |  | -.28 | .197 | |  | .041 | .713 |  | -.188 | .273 |
| AMP |  | -.143 | .219 |  | -2.986 | .695 |  | -.132 | .251 |  | -2.887 | .768 | |  | -.153 | .163 |  | -2.964 | .51 |
| cAMP |  | -.038 | .744 |  | -44.048 | 31.593 |  | -.003 | .978 |  | -38.28 | 37.222 | |  | -.026 | .814 |  | -40.105 | 31.603 |
| Cytidine |  | .015 | .901 |  | -3.769 | 4.274 |  | -.009 | .936 |  | -4.136 | 3.814 | |  | -.069 | .542 |  | -5.118 | 2.713 |
| Guanosine |  | -.002 | .983 |  | -.309 | .303 |  | -.104 | .399 |  | -.46 | .185 | |  | -.048 | .664 |  | -.352 | .225 |
| IMP |  | -.066 | .574 |  | -1.896 | 1.058 |  | -.091 | .436 |  | -2.052 | .894 | |  | -.073 | .508 |  | -1.878 | .939 |
| Inosine |  | .056 | .632 |  | -.082 | .134 |  | -.009 | .942 |  | -.114 | .106 | |  | .026 | .814 |  | -.09 | .114 |
| Xanthosine |  | .04 | .73 |  | -4.288 | 6.089 |  | .033 | .785 |  | -4.691 | 6.182 | |  | .004 | .969 |  | -4.915 | 5.111 |
|  |  |  |  |  |  |  |  |  |  |  |  |  |  |  |  |  |  |  |  |
| **Nucleobases** | | | |  |  |  |  |  |  |  |  |  |  |  |  |  |  |  |  |
|  |  |  |  |  |  |  |  |  |  |  |  |  |  |  |  |  |  |  |  |
| Adenine |  | -.095 | .414 |  | -113.099 | 47.015 |  | -.053 | .648 |  | -98.094 | 61.375 | |  | -.085 | .436 |  | -104.755 | 45.623 |
| Cytosine |  | -.065 | .579 |  | -20.691 | 11.642 |  | -.078 | .512 |  | -21.946 | 11.034 | |  | -.044 | .693 |  | -18.382 | 12.286 |
| Hypoxanthine | | -.077 | .509 |  | -.049 | .024 |  | -.067 | .56 |  | -.047 | .025 | |  | -.132 | .23 |  | -.056 | .014 |
| Neopterin |  | .077 | .509 |  | -53.440 | 106.734 |  | .043 | .71 |  | -64.598 | 94.353 | |  | .109 | .32 |  | -37.564 | 113.497 |
| Uracil |  | -.063 | .586 |  | -12.433 | 7.079 |  | -.033 | .779 |  | -11.16 | 8.4 | |  | .009 | .935 |  | -8.987 | 9.758 |
| Xanthine |  | -.012 | .917 |  | -.398 | .358 |  | -.04 | .734 |  | -.45 | .319 | |  | -.119 | .291 |  | -.559 | .17 |
|  |  |  |  |  |  |  |  |  |  |  |  |  |  |  |  |  |  |  |  |
| **Organic compounds, carbohydrates, and carbohydrate conjugates** | | | | | | | | |  |  |  |  |  |  |  |  |  |  |  |
|  |  |  |  |  |  |  |  |  |  |  |  |  |  |  |  |  |  |  |  |
| 1-Methylhistamine | | .169 | .144 |  | -23.016 | 155.051 |  | .058 | .651 |  | -77.203 | 122.781 | |  | .092 | .41 |  | -50.492 | 122.244 |
| Trimethylamine-N-oxide | | .082 | .482 |  | -.029 | .060 |  | .074 | .519 |  | -.03 | .058 | |  | .027 | .805 |  | -.037 | .048 |
| D-Glucuronic acid | | .225 | .051 |  | -.001 | .596 |  | .174 | .138 |  | -.076 | .535 | |  | .186 | .088 |  | -.037 | .529 |
| Glyceraldehyde | | .063 | .59 |  | -.002 | .004 |  | .001 | .991 |  | -.004 | .004 | |  | .037 | .733 |  | -.003 | .004 |
| Hippuric acid | | -.123 | .292 |  | -.091 | .028 |  | -.135 | .246 |  | -.094 | .025 | |  | -.109 | .326 |  | -.085 | .029 |
| 3-Hydroxyanthranilic acid | | -.044 | .707 |  | -4.718 | 3.214 |  | -.043 | .708 |  | -4.633 | 3.163 | |  | -.022 | .844 |  | -4.099 | 3.36 |
| Myoinositol |  | .026 | .823 |  | -.093 | .116 |  | .064 | .593 |  | -.078 | .136 | |  | .089 | .423 |  | -.059 | .14 |
| D-Ribose 5-phosphate | | -.034 | .773 |  | -.22 | .016 |  | -.025 | .828 |  | -.021 | .017 | |  | .022 | .844 |  | -.016 | .02 |
| Sucrose |  | -.206 | .074 |  | -.040 | .002 |  | -.189 | .099 |  | -.039 | .003 | |  | -.167 | .127 |  | -.036 | .005 |
|  |  |  |  |  |  |  |  |  |  |  |  |  |  |  |  |  |  |  |  |
| **Bile acids** | | | |  |  |  |  |  |  |  |  |  |  |  |  |  |  |  |  |
|  |  |  |  |  |  |  |  |  |  |  |  |  |  |  |  |  |  |  |  |
| Chenodeoxycholic acid | | -.063 | .586 |  | -.011 | .006 |  | -.059 | .61 |  | -.011 | .006 | |  | -.036 | .744 |  | -.01 | .007 |
| Cholic acid |  | -.077 | .511 |  | -.027 | .014 |  | -.124 | .283 |  | -.031 | .009 | |  | -.03 | .783 |  | -.022 | .017 |
| Glycocholic acid | | -.094 | .417 |  | -.597 | .250 |  | -.048 | .68 |  | -.512 | .336 | |  | -.058 | .599 |  | -.508 | .295 |
| Taurine |  | -.015 | .897 |  | -.004 | .004 |  | -.104 | .412 |  | -.006 | .002 | |  | -.038 | .737 |  | -.004 | .003 |
| Taurochenodeoxycholic acid | | -.022 | .852 |  | -.202 | .167 |  | -.018 | .88 |  | -.201 | .172 | |  | -.032 | .77 |  | -.199 | .148 |
| Taurocholic acid | | .028 | .813 |  | -2.225 | 2.826 |  | -.01 | .933 |  | -2.618 | 2.406 | |  | -.044 | .698 |  | -2.924 | 1.969 |
|  |  |  |  |  |  |  |  |  |  |  |  |  |  |  |  |  |  |  |  |
| **Enzyme cofactors** | | | |  |  |  |  |  |  |  |  |  |  |  |  |  |  |  |  |
|  |  |  |  |  |  |  |  |  |  |  |  |  |  |  |  |  |  |  |  |
| 4-Pyridoxic acid | | -.016 | .892 |  | -3.906 | 3.407 |  | -.086 | .466 |  | -5.031 | 2.327 | |  | -.021 | .849 |  | -3.778 | 3.116 |
| Glutathione |  | -.14 | .226 |  | -3.582 | .862 |  | -.081 | .494 |  | -3.061 | 1.49 | |  | -.086 | .44 |  | -2.971 | 1.304 |
| NAD |  | -.079 | .496 |  | -14.802 | 68.836 |  | -.097 | .4 |  | -147.84 | 59.709 | |  | -.074 | .503 |  | -133.866 | 66.236 |
| Niacinamide | | -.17 | .143 |  | -1.066 | .157 |  | -.177 | .122 |  | -1.076 | .13 | |  | -.116 | .31 |  | -.916 | .295 |
| Nicotinic acid | | .105 | .367 |  | -97.651 | 260.977 |  | .156 | .175 |  | -55.401 | 298.79 | |  | .135 | .22 |  | -63.867 | 273.478 |
| Pantothenic acid | | .068 | .559 |  | -1.039 | 1.905 |  | -.061 | .637 |  | -2.039 | 1.256 | |  | .055 | .617 |  | -1.039 | 1.738 |
|  |  |  |  |  |  |  |  |  |  |  |  |  |  |  |  |  |  |  |  |
| **Neurotransmitter metabolic intermediates** | | | | | | |  |  |  |  |  |  |  |  |  |  |  |  |  |
|  |  |  |  |  |  |  |  |  |  |  |  |  |  |  |  |  |  |  |  |
| GABA |  | -.121 | .299 |  | -1.471 | .458 |  | -.125 | .274 |  | -1.477 | .425 | |  | -.092 | .402 |  | -1.301 | .528 |
| L-5-Hydroxytryptophan | | .027 | .818 |  | -6.484 | 8.182 |  | -.021 | .865 |  | -8.325 | 7.011 | |  | .028 | .798 |  | -5.993 | 7.766 |
| Normetanephrine | | .095 | .415 |  | -24.898 | 59.737 |  | .015 | .904 |  | -42.582 | 48.092 | |  | .085 | .452 |  | -25.656 | 56.964 |
|  |  |  |  |  |  |  |  |  |  |  |  |  |  |  |  |  |  |  |  |

Legend: *B*, standardized regression coefficient; *p*, statistical significance; 95% CI, 95% confidence interval; Adjusted model 1 included as covariates BMI (body mass index), ASSIST Tobacco (smoking), and AUDIT-C (alcohol consumption); Adjusted model 2 included as covariates BDI (Beck Depression Inventory) and depression chronicity. Statistically significant metabolites in linear or logistic regression are bolded.
