# Supplementary material for: The associations between metabolic profiles and sexual and physical abuse in depressed adolescent psychiatric outpatients: an exploratory pilot study
Source: Eur J Psychotraumatol. 2023 Mar 29;14(1):2191396. doi: 10.1080/20008066.2023.2191396 (PMC10062226; doi:10.1080/20008066.2023.2191396)
Supplement: Supplemental Material [file ZEPT_A_2191396_SM0818.docx]

Supplementary Table 2, Linear regression coefficients with Trauma and Distress Scale (TADS) Physical Abuse factor scores across metabolites, adjusting for selected covariates in Models 1 and 2.

|  |  | Unadjusted model | | | | |  | Adjusted model 1 | | | | |  | Adjusted model 2 | | | | |
| --- | --- | --- | --- | --- | --- | --- | --- | --- | --- | --- | --- | --- | --- | --- | --- | --- | --- | --- |
|  |  | *B* | *p* |  | 95% CI | |  | *B* | *p* |  | 95% CI | |  | *B* | *p* |  | 95% CI | |
|  |  |  |  |  | Lower bound | Upper bound | |  |  |  | Lower bound | Upper bound | |  |  |  | Lower bound | Upper bound |
| **Choline and mitochondrial metabolites** | | | |  |  |  |  |  |  |  |  |  |  |  |  |  |  |  |
|  |  |  |  |  |  |  |  |  |  |  |  |  |  |  |  |  |  |  |
| Acetoacetic acid | | -.104 | .373 |  | -.002 | .001 |  | -.058 | .62 |  | -.001 | .001 |  | -.051 | .661 |  | -.001 | .001 |
| Allantoin |  | .032 | .784 |  | -.190 | .250 |  | .077 | .499 |  | -.141 | .288 |  | .028 | .797 |  | -.182 | .236 |
| Glycine betaine | | -.047 | .684 |  | -.006 | .004 |  | -.023 | .839 |  | -.005 | .004 |  | .025 | .824 |  | -.004 | .005 |
| Carnitine |  | .135 | .243 |  | -.009 | .035 |  | .132 | .252 |  | -.009 | .034 |  | .099 | .381 |  | -.012 | .03 |
| Carnosine |  | .037 | .75 |  | -33.831 | 46.758 |  | .041 | .719 |  | -32.125 | 46.332 |  | -.01 | .928 |  | -40.304 | 36.819 |
| **Choline** |  | **-.276** | **.016** |  | **-.091** | **-.010** |  | **-.292** | **.009** |  | **-.093** | **-.014** |  | -.182 | .115 |  | -.075 | .008 |
| Cotinine |  | .227 | .265 |  | -.268 | .929 |  | .307 | .143 |  | -.040 | .260 |  | .047 | .831 |  | -.928 | 1.143 |
| **Gamma-glutamyl cysteine** | | -.201 | .082 |  | -.326 | .020 |  | **-.236** | **.036** |  | **-.347** | **-.012** |  | **-.231** | **.035** |  | **-.339** | **-.013** |
| Phosphoethanolamine | | -.027 | .819 |  | -.641 | .509 |  | -.008 | .945 |  | -.579 | .541 |  | .042 | .708 |  | -.449 | .658 |
| Spermidine |  | .136 | .243 |  | -1.553 | 6.033 |  | .104 | .37 |  | -2.082 | 5.522 |  | .143 | .194 |  | -1.231 | 5.957 |
| **Succinate** |  | **-.233** | **.042** |  | **-.092** | **-.002** |  | -.214 | .072 |  | -.09 | .004 |  | -.156 | .173 |  | -.077 | .014 |
|  |  |  |  |  |  |  |  |  |  |  |  |  |  |  |  |  |  |  |
| **Nucleotides and nucleosides** | | | |  |  |  |  |  |  |  |  |  |  |  |  |  |  |  |
|  |  |  |  |  |  |  |  |  |  |  |  |  |  |  |  |  |  |  |
| Deoxycytidine | | .198 | .086 |  | -.078 | 1.139 |  | .141 | .245 |  | -.265 | 1.019 |  | .139 | .222 |  | -.231 | .977 |
| 2-Deoxyuridine | | .044 | .707 |  | -7.541 | 11.065 |  | .035 | .761 |  | -7.71 | 10.502 |  | .03 | .785 |  | -7.61 | 10.037 |
| Adenosine |  | -.001 | .991 |  | -.233 | .231 |  | .003 | .978 |  | -.225 | .231 |  | .048 | .674 |  | -.177 | .272 |
| **AMP** |  | **-.248** | **.031** |  | **-3.661** | **-.185** |  | -.211 | .062 |  | -3.348 | .083 |  | **-.283** | **.009** |  | **-3.83** | **-.56** |
| cAMP |  | -.1 | .39 |  | -52.085 | 2.576 |  | -.05 | .663 |  | -43.821 | 28.044 |  | -.067 | .549 |  | -45.362 | 24.308 |
| Cytidine |  | .081 | .489 |  | -2.516 | 5.218 |  | .072 | .525 |  | -2.567 | 4.989 |  | .052 | .653 |  | -2.954 | 4.68 |
| Guanosine |  | .017 | .881 |  | -.273 | .317 |  | -.119 | .327 |  | -.459 | .155 |  | -.028 | .804 |  | -.317 | .246 |
| IMP |  | .04 | .731 |  | -1.180 | 1.674 |  | -.017 | .884 |  | -1.514 | 1.306 |  | .004 | .972 |  | -1.351 | 1.4 |
| Inosine |  | .049 | .677 |  | -.082 | .126 |  | -.04 | .734 |  | -.123 | .087 |  | .012 | .917 |  | -.094 | .105 |
| Xanthosine |  | -.053 | .649 |  | -6.151 | 3.853 |  | -.021 | .864 |  | -5.63 | 4.736 |  | -.128 | .259 |  | -7.6 | 2.076 |
|  |  |  |  |  |  |  |  |  |  |  |  |  |  |  |  |  |  |  |
| **Organic compounds, carbohydrates, and carbohydrate conjugates** | | | | | | | | |  |  |  |  |  |  |  |  |  |  |
|  |  |  |  |  |  |  |  |  |  |  |  |  |  |  |  |  |  |  |
| 1-Methylhistamine | | .161 | .163 |  | -25.248 | 146.771 |  | .038 | .764 |  | -80.941 | 109.819 |  | .093 | .409 |  | -49.059 | 119.121 |
| Trimethylamine-N-oxide | | .172 | .138 |  | -.010 | .074 |  | .148 | .19 |  | -.014 | .069 |  | .11 | .326 |  | -.021 | .061 |
| **D-Glucuronic acid** | | **.233** | **.043** |  | **.009** | **.584** |  | .167 | .149 |  | -.078 | .504 |  | .203 | .064 |  | -.016 | .534 |
| Glyceraldehyde | | -.091 | .432 |  | -.004 | .002 |  | -.185 | .14 |  | -.006 | .001 |  | -.105 | .34 |  | -.004 | .002 |
| Hippuric acid | | -.05 | .665 |  | -.070 | .045 |  | -.08 | .487 |  | -.077 | .037 |  | -.068 | .541 |  | -.073 | .038 |
| 3-Hydroxyanthranilic acid | | -.177 | .125 |  | -6.703 | .836 |  | -.175 | .119 |  | -6.549 | .762 |  | -.155 | .157 |  | -6.152 | 1.012 |
| Myoinositol |  | -.091 | .436 |  | -.140 | .061 |  | -.006 | .958 |  | -.105 | .1 |  | -.025 | .825 |  | -.108 | .086 |
| D-Ribose 5-phosphate | | .002 | .987 |  | -.018 | .019 |  | .01 | .93 |  | -.017 | .019 |  | .039 | .725 |  | -.015 | .021 |
| Sucrose |  | -.176 | .128 |  | -.036 | .005 |  | -.159 | .16 |  | -.034 | .006 |  | -.153 | .165 |  | -.033 | .006 |
|  |  |  |  |  |  |  |  |  |  |  |  |  |  |  |  |  |  |  |
| **Amino acids and derivatives** | | | |  |  |  |  |  |  |  |  |  |  |  |  |  |  |  |
|  |  |  |  |  |  |  |  |  |  |  |  |  |  |  |  |  |  |  |
| Aminoadipic acid | | -.071 | .543 |  | -.282 | .150 |  | -.12 | .296 |  | -.324 | .1 |  | -.094 | .402 |  | -.297 | .121 |
| Aminoisobutyric acid | | -.05 | .667 |  | -3.440 | 2.215 |  | -.049 | .672 |  | -3.404 | 2.207 |  | -.101 | .362 |  | -3.927 | 1.452 |
| Alanine |  | .051 | .659 |  | -.001 | .002 |  | .004 | .972 |  | -.002 | .002 |  | .12 | .286 |  | -.001 | .003 |
| Arginine |  | .02 | .864 |  | -.009 | .011 |  | -.009 | .935 |  | -.011 | .01 |  | -.009 | .933 |  | -.01 | .009 |
| Asparagine |  | -.177 | .127 |  | -.031 | .004 |  | -.171 | .134 |  | -.03 | .004 |  | -.142 | .21 |  | -.028 | .006 |
| Asymmetric dimethylarginine | | -.1 | .39 |  | -.779 | .307 |  | -.134 | .244 |  | -.852 | .22 |  | -.136 | .219 |  | -.839 | .196 |
| Citrulline |  | -.075 | .522 |  | -.032 | .017 |  | -.046 | .685 |  | -.029 | .019 |  | -.146 | .195 |  | -.039 | .008 |
| Creatine |  | .07 | .549 |  | -.005 | .008 |  | -.084 | .503 |  | -.009 | .005 |  | .004 | .97 |  | -.006 | .006 |
| Creatinine |  | 0 | .998 |  | -.009 | .009 |  | -.011 | .928 |  | -.009 | .009 |  | .041 | .714 |  | -.007 | .01 |
| Cystathionine | | .181 | .117 |  | -2.290 | 20.179 |  | .186 | .113 |  | -2.222 | 20.569 |  | .041 | .204 |  | -3.843 | 17.706 |
| Dimethylglycine | | -.061 | .601 |  | -.112 | .065 |  | -.071 | .535 |  | -.114 | .06 |  | -.124 | .266 |  | -.132 | .037 |
| Glutamate | | .117 | .312 |  | -.009 | .026 |  | .007 | .957 |  | -.018 | .019 |  | .014 | .906 |  | -.017 | .019 |
| Glutamine |  | .046 | .692 |  | -.001 | .001 |  | .088 | .457 |  | 0 | .001 |  | .122 | .278 |  | 0 | .001 |
| Glycine |  | .011 | .928 |  | -.001 | .001 |  | .077 | .52 |  | -.001 | .002 |  | .072 | .523 |  | -.001 | .002 |
| Guanidinoacetic acid | | -.013 | .908 |  | -.249 | .221 |  | .082 | .5 |  | -.161 | .327 |  | .055 | .628 |  | -.172 | .283 |
| Histidine |  | -.174 | .133 |  | -.013 | .002 |  | -.188 | .095 |  | -.013 | .001 |  | -.207 | .059 |  | -.013 | 0 |
| Homocysteine | | -.019 | .872 |  | -.886 | .753 |  | .034 | .769 |  | -.689 | .927 |  | .026 | .817 |  | -.699 | .884 |
| Homogentisic acid | | .138 | .236 |  | -.818 | 3.268 |  | .098 | .41 |  | -1.224 | 2.965 |  | .091 | .413 |  | -1.152 | 2.775 |
| Hydroxyproline | | .011 | .924 |  | -.023 | .025 |  | .005 | .963 |  | -.023 | .024 |  | -.029 | .81 |  | -.028 | .022 |
| Isoleucine |  | .197 | .088 |  | .000 | .007 |  | .096 | .432 |  | -.002 | .005 |  | .151 | .171 |  | -.001 | .006 |
| Kynurenic acid | | -.208 | .072 |  | -31.514 | 1.366 |  | -.227 | .053 |  | -33.143 | .228 |  | -.154 | .169 |  | -27.146 | 4.844 |
| L-Kynurenine | | .052 | .654 |  | -.851 | 1.347 |  | .069 | .558 |  | -.779 | 1.431 |  | .051 | .646 |  | -.799 | 1.28 |
| Leucine |  | .122 | .295 |  | -.003 | .009 |  | .02 | .87 |  | -.006 | .007 |  | .079 | .481 |  | -.004 | .008 |
| Lysine |  | -.02 | .861 |  | -.005 | .004 |  | -.144 | .24 |  | -.008 | .002 |  | -.022 | .843 |  | -.005 | .004 |
| L-Methionine | | -.025 | .832 |  | -.023 | .019 |  | -.019 | .87 |  | -.022 | .019 |  | -.006 | .959 |  | -.02 | .019 |
| Ornithine |  | .132 | .257 |  | -.005 | .018 |  | .101 | .388 |  | -.007 | .017 |  | .095 | .39 |  | -.006 | .016 |
| Phenylalanine | | .032 | .785 |  | -.009 | .012 |  | -.044 | .716 |  | -.013 | .009 |  | .003 | .981 |  | -.01 | .01 |
| Proline |  | .01 | .935 |  | -.003 | .003 |  | .04 | .722 |  | -.002 | .004 |  | -.006 | .956 |  | -.003 | .003 |
| Serine |  | -.056 | .63 |  | -.007 | .004 |  | -.012 | .916 |  | -.006 | .006 |  | -.004 | .969 |  | -.006 | .006 |
| Symmetric dimethylarginine | | -.037 | .753 |  | -.472 | .343 |  | -.053 | .638 |  | -.492 | .303 |  | -.066 | .551 |  | -.504 | .271 |
| Threonine |  | -.187 | .105 |  | -.007 | .001 |  | -.163 | .154 |  | -.006 | .001 |  | -.153 | .167 |  | -.006 | .001 |
| Tryptophan |  | .037 | .75 |  | -.038 | .052 |  | .033 | .773 |  | -.038 | .05 |  | .054 | .628 |  | -.032 | .053 |
| Tyrosine |  | .107 | .359 |  | -.008 | .022 |  | .064 | .58 |  | -.011 | .019 |  | .111 | .311 |  | -.007 | .021 |
| Valine |  | .098 | .398 |  | -.001 | .002 |  | .003 | .981 |  | -.001 | .001 |  | .079 | .474 |  | -.001 | .002 |
|  |  |  |  |  |  |  |  |  |  |  |  |  |  |  |  |  |  |  |
| **Acylcarnitines** | | | |  |  |  |  |  |  |  |  |  |  |  |  |  |  |  |
|  |  |  |  |  |  |  |  |  |  |  |  |  |  |  |  |  |  |  |
| Acetylcarnitine | | .11 | .345 |  | -.032 | .090 |  | .079 | .489 |  | -.039 | .081 |  | .096 | .408 |  | -.035 | .086 |
| Propionylcarnitine | | .17 | .142 |  | -5.580 | 38.092 |  | .119 | .329 |  | -11.678 | 34.376 |  | .105 | .356 |  | -11.556 | 31.723 |
| Isobutyrylcarnitine | | -.002 | .985 |  | -16.786 | 16.462 |  | -.019 | .869 |  | -17.511 | 14.828 |  | -.036 | .749 |  | -18.558 | 13.4 |
| Isovalerylcarnitine | | .157 | .175 |  | -3.613 | 19.538 |  | .111 | .355 |  | -6.399 | 17.622 |  | .106 | .341 |  | -5.801 | 16.565 |
| Hexanoylcarnitine | | -.02 | .864 |  | -31.181 | 26.213 |  | -.055 | .64 |  | -35.964 | 22.241 |  | .033 | .771 |  | -23.919 | 32.14 |
| Octanoylcarnitine | | -.112 | .335 |  | -4.679 | 1.614 |  | -.082 | .487 |  | -4.346 | 2.092 |  | -.047 | .685 |  | -3.807 | 2.515 |
| Decanoylcarnitine | | -.093 | .423 |  | -7.928 | 3.366 |  | -.044 | .706 |  | -6.771 | 4.611 |  | -.054 | .641 |  | -6.952 | 4.307 |
|  |  |  |  |  |  |  |  |  |  |  |  |  |  |  |  |  |  |  |
| **Nucleobases** | | | |  |  |  |  |  |  |  |  |  |  |  |  |  |  |  |
|  |  |  |  |  |  |  |  |  |  |  |  |  |  |  |  |  |  |  |
| Adenine |  | -.066 | .57 |  | -99.611 | 55.222 |  | -.03 | .796 |  | -85.96 | 66.185 |  | -.065 | .558 |  | -95.015 | 51.67 |
| Cytosine |  | -.144 | .213 |  | -25.213 | 5.718 |  | -.105 | .369 |  | -22.78 | 8.57 |  | -.152 | .171 |  | -24.991 | 4.512 |
| Hypoxanthine | | -.11 | .342 |  | -.052 | .018 |  | -.101 | .371 |  | -.05 | .019 |  | -.148 | .181 |  | -.056 | .011 |
| Neopterin |  | .059 | .614 |  | -57.700 | 97.019 |  | .015 | .892 |  | -70.64 | 80.986 |  | .105 | .343 |  | -38.357 | 108.818 |
| Uracil |  | -.042 | .72 |  | -11.121 | 7.724 |  | -.023 | .845 |  | -10.241 | 8.407 |  | .034 | .761 |  | -7.723 | 10.517 |
| Xanthine |  | .066 | .568 |  | -.259 | .469 |  | 0 | .997 |  | -.366 | .367 |  | -.024 | .832 |  | -.396 | .319 |
|  |  |  |  |  |  |  |  |  |  |  |  |  |  |  |  |  |  |  |
| **Bile acids** | | | |  |  |  |  |  |  |  |  |  |  |  |  |  |  |  |
|  |  |  |  |  |  |  |  |  |  |  |  |  |  |  |  |  |  |  |
| Chenodeoxycholic acid | | .004 | .976 |  | -.008 | .009 |  | .006 | .959 |  | -.008 | .009 |  | .015 | .89 |  | -.008 | .009 |
| CholicAcid |  | -.083 | .478 |  | -.027 | .013 |  | -.118 | .301 |  | -.029 | .009 |  | -.038 | .734 |  | -.022 | .016 |
| Glycocholic acid | | -.111 | .338 |  | -.605 | .210 |  | -.087 | .446 |  | -.557 | .248 |  | -.067 | .546 |  | -.51 | .272 |
| Taurine |  | .073 | .513 |  | -.003 | .005 |  | -.008 | .952 |  | -.004 | .004 |  | .022 | .848 |  | -.003 | .004 |
| Taurochenodeoxycholic acid | | .078 | .502 |  | -.118 | .238 |  | .044 | .706 |  | -.144 | .211 |  | .069 | .532 |  | -.115 | .221 |
| Taurocholic acid | | .084 | .472 |  | -1.548 | 3.309 |  | .066 | .566 |  | -1.698 | 3.079 |  | .062 | .589 |  | -1.732 | 3.027 |
|  |  |  |  |  |  |  |  |  |  |  |  |  |  |  |  |  |  |  |
| **Enzyme cofactors** | | | |  |  |  |  |  |  |  |  |  |  |  |  |  |  |  |
|  |  |  |  |  |  |  |  |  |  |  |  |  |  |  |  |  |  |  |
| 4-Pyridoxic acid | | .12 | .301 |  | -1.673 | 5.331 |  | .071 | .54 |  | -2.425 | 4.595 |  | .099 | .369 |  | -1.825 | 4.852 |
| Glutathione |  | -.088 | .448 |  | -2.982 | 1.331 |  | -.038 | .749 |  | -2.525 | 1.824 |  | -.065 | .564 |  | -2.692 | 1.479 |
| NAD |  | .076 | .515 |  | -67.916 | 134.385 |  | .037 | .745 |  | -83.036 | 115.626 |  | .052 | .644 |  | -74.83 | 120.319 |
| Niacinamide | | -.126 | .277 |  | -.920 | .268 |  | -.136 | .231 |  | -.929 | .228 |  | -.127 | .271 |  | -.916 | .261 |
| Nicotinic acid | | .058 | .618 |  | -130.002 | 217.316 |  | .106 | .355 |  | -90.659 | 249.29 |  | .1 | .366 |  | -89.688 | 240.356 |
| Pantothenic acid | | .221 | .055 |  | -.029 | 2.747 |  | .122 | .342 |  | -.813 | 2.312 |  | .195 | .075 |  | -.124 | 2.526 |
|  |  |  |  |  |  |  |  |  |  |  |  |  |  |  |  |  |  |  |
| **Neurotransmitter metabolic intermediates** | | | |  |  |  |  |  |  |  |  |  |  |  |  |  |  |  |
|  |  |  |  |  |  |  |  |  |  |  |  |  |  |  |  |  |  |  |
| GABA |  | -.191 | .099 |  | -1.691 | .149 |  | -.179 | .112 |  | -1.623 | .173 |  | -.151 | .171 |  | -1.494 | .271 |
| L-5-Hydroxytryptophan | | -.038 | .743 |  | -8.242 | 5.901 |  | -.156 | .192 |  | -11.993 | 2.452 |  | -.032 | .77 |  | -7.685 | 5.71 |
| Normetanephrine | | .189 | .102 |  | -6.837 | 73.710 |  | .121 | .323 |  | -21.512 | 64.323 |  | .139 | .223 |  | -15.315 | 64.611 |
|  |  |  |  |  |  |  |  |  |  |  |  |  |  |  |  |  |  |  |

Legend: B, standardized regression coefficient; *p*, statistical significance; 95% CI, 95% confidence interval; Adjusted model 1 included as covariates BMI (body mass index), ASSIST Tobacco (smoking), and AUDIT-C (alcohol consumption); Adjusted model 2 included as covariates BDI (Beck Depression Inventory) and depression chronicity. Statistically significant metabolites in linear or logistic regression are bolded.
