# Supplementary material for: The associations between metabolic profiles and sexual and physical abuse in depressed adolescent psychiatric outpatients: an exploratory pilot study
Source: Eur J Psychotraumatol. 2023 Mar 29;14(1):2191396. doi: 10.1080/20008066.2023.2191396 (PMC10062226; doi:10.1080/20008066.2023.2191396)
Supplement: Supplemental Material [file ZEPT_A_2191396_SM0797.docx]

| Supplementary Table 3.  Standardized loadings and thresholds of the confirmatory five-dimensional TADS factor model. | | | | | | |
| --- | --- | --- | --- | --- | --- | --- |
| Item | | Loading | Thresholds | | | |
|  |  |  | 1 | 2 | 3 | 4 |
| Emotional Neglect | |  |  |  |  |  |
| 5 | I felt valued (liked) or important | -.68 | -1.52 | -.68 | .18 | 1.01 |
| 8 | My family was emotionally warm and loving | -.92 | -1.79 | -1.20 | -.39 | .39 |
| 13 | My family took care of each other | -.89 | -2.04 | -1.28 | -.61 | .31 |
| 21 | I respect myself | -.48 | -1.36 | -.39 | .49 | 1.40 |
| 40 | My family was supportive and encouraging | -.95 | -1.82 | -1.10 | -.34 | .48 |
| Physical Neglect | |  |  |  |  |  |
| 1 | I felt safe and protected by someone | -.82 | -1.89 | -1.09 | -.33 | .47 |
| 2 | I was often hungry | .38 | -.06 | .72 | 1.58 | 2.15 |
| 4 | I often wore ragged or dirty clothes in school | .47 | .80 | 1.53 | 2.09 | 2.47 |
| 6 | My parents/caregivers were often drunk, stoned or wasted | .37 | .24 | .72 | 1.36 | 2.15 |
| 31 | If I needed treatment, someone would always take me to see a doctor or nurse | -.47 | -1.60 | -1.29 | -.79 | -.02 |
| Emotional Abuse | |  |  |  |  |  |
| 10 | I felt rejected by my parents/caregivers | .85 | .14 | .66 | 1.41 | 1.92 |
| 12 | I was humiliated by persons in my family | .78 | .15 | .58 | 1.32 | 1.92 |
| 14 | I believe that I am a bad person | .52 | -.42 | .19 | 1.00 | 1.56 |
| 26 | I felt hated by a member or members of my family | .80 | .05 | .58 | 1.29 | 1.92 |
| 32 | I feel that I was put down, criticized, and made to feel inferior | .76 | -.26 | .22 | .84 | 1.62 |
| Physical Abuse | |  |  |  |  |  |
| 9 | I was hit so hard that it left marks, cuts, or bruises | .83 | .57 | .93 | 1.83 | 2.36 |
| 16 | I have experienced serious physical assault | .90 | .84 | 1.17 | 1.92 | 2.61 |
| 17 | Adults noticed cuts, bruises, or marks from when I was beaten | .52 | 1.34 | 1.62 | 2.20 | 2.27 |
| 20 | I think I was physically abused | .89 | .64 | 1.08 | 1.58 | 2.04 |
| 24 | I have been involved in life-threatening situations | .66 | .34 | 1.02 | 1.92 | 1.32 |
| Sexual Abuse | |  |  |  |  |  |
| 22 | When I was young, someone touched me or tried to make me touch him/her in a sexual way | .80 | 1.32 | 1.56 | 1.92 | 2.36 |
| 25 | I was forced to keep secrets about someone sexually interfering with me | .93 | 1.52 | 1.74 | 2.20 | 2.28 |
| 30 | I have experienced sexual assault | .92 | .86 | 1.24 | 1.85 | 2.21 |
| 33 | Someone sexually molested me | .96 | .68 | 1.07 | 1.62 | 2.27 |
| 41 | I believe that I was sexually abused | .93 | 1.03 | 1.39 | 1.82 | 2.28 |
| Note: Items with negative loadings are reverse-worded, including all items on Emotional Neglect. | | | | | | |
