# Supplementary material for: The associations between metabolic profiles and sexual and physical abuse in depressed adolescent psychiatric outpatients: an exploratory pilot study
Source: Eur J Psychotraumatol. 2023 Mar 29;14(1):2191396. doi: 10.1080/20008066.2023.2191396 (PMC10062226; doi:10.1080/20008066.2023.2191396)
Supplement: Supplemental Material [file ZEPT_A_2191396_SM0772.docx]

Supplementary Table 4.
Factor correlations of the five-dimensional TADS factor model.

|  | Emotional Neglect | Physical Neglect | Emotional Abuse | Physical Abuse | Sexual Abuse |
| --- | --- | --- | --- | --- | --- |
| Emotional Neglect | 1 |  |  |  |  |
| Physical Neglect | .92 | 1 |  |  |  |
| Emotional Abuse | .83 | .85 | 1 |  |  |
| Physical Abuse | .55 | .77 | .68 | 1 |  |
| Sexual Abuse | .30 | .41 | .53 | .56 | 1 |
